# General practitioners treating their own family members: a cross-sectional survey in Germany

**DOI:** 10.1186/s12875-022-01631-z

**Published:** 2022-02-03

**Authors:** Natalie Alida Mücke, Alexandra Schmidt, Christine Kersting, Vera Kalitzkus, Michael Pentzek, Stefan Wilm, Achim Mortsiefer

**Affiliations:** 1 Institute of General Practice, Medical Faculty, Heinrich-Heine-University Düsseldorf, Düsseldorf, Germany; 2 Chair of General Practice II and Patient-Centredness in Primary Care, Institute of General Practice and Primary Care, Faculty of Health, Witten/Herdecke University, Witten, Germany

**Keywords:** General practice, Family medicine, Primary care, Informal medicine

## Abstract

**Background:**

In Germany, there are neither guidelines provided by the medical associations nor a public discussion about general practitioners (GPs) treating their family members. Only few studies on this topic from the primary care setting exist. The aim of this study is to describe GPs’ treatment of family members and to generate empirical data on the most common reasons for this.

**Methods:**

In June 2018 we conducted a postal survey among GPs in the North Rhine region of Germany. The questionnaire was developed in a stepwise process including initial expert panels, interviews with GPs, item construction workshops, cognitive pre-tests and pilot testing with 40 questionnaires. The final questionnaire addressed: type and frequency of treatment, documentation and place of treatment, engagement as the official GP as well as reasons for and against the treatment. For data evaluation, descriptive and explorative statistical analyses were conducted.

**Results:**

Overall, 393 questionnaires were returned (response rate 39.8%). 96.7% of the GPs had treated at least one family member during the last 12 months. Services that were provided frequently (more than three times in the last 12 months) included the prescription or dispensing of medication (partner 45%, children 37%, parents 43%, partner’s parents 26%), physical examinations (partner 18%, children 24%, parents 25%, partner’s parents 15%), and the arrangement or provision of laboratory tests (partner 14%, children 7%, parents 16%, partner’s parents 9%). Less than one third of the study participants always treated their relatives in their office. Male GPs more often provided care to family members (except children) registered in their practice. Senior male GPs treated their relatives more often than junior female GPs. Family members were most commonly treated for practical reasons.

**Conclusion:**

The subject of GPs treating their relatives is of high everyday relevance, since nearly all GPs are involved in the treatment of their family members. Frequent at-home treatments and low documentation rates may indicate risks due to deviating from the professional routine.

## Background

In Germany, there is neither an explicit legal basis nor a professional guideline for treating family members; at the same time, providing medical treatment to one’s relatives is not forbidden. In fact, even prescribing anaesthetics and performing surgical procedures is permitted [1].

According to § 12 (3) of the (Model) Professional Code for Physicians of the German Medical Association (2018), physicians are permitted to treat their relatives at no cost or at a reduced fee [2]. Irrespectively of receiving payment, all treatments are covered by the professional liability insurance. Informal treatments of relatives without payment do not even have to be performed in the GP’s office in order for the costs to be covered.

Unlike Germany, in many other countries the topic of treating family members is the subject of controversial debate, and clear recommendations are often provided. In the United States, the Code of Medical Ethics of the American Medical Association [3] clearly recommends not providing any treatment to relatives. For physicians in Australia, the Medical Board of Australia (2014) states: “Whenever possible, avoid providing medical care to anyone with whom you have a close personal relationship” [4]. In Brazil, medical practice has since 1932 been regulated by a decree that explicitly prohibits the treatment of family members of the same household suffering from a serious illness or addiction. Exceptions are made for regions where physicians are scarce [5]. Similar recommendations and views exist in the United Kingdom, Canada, Norway, and the Netherlands [6–10]. However, none of the international recommendations for treating own relatives refer to emergency situations, where treatment should be provided without hesitation [3, 4, 6, 7, 11, 12].

Taking a specific look at GPs reveals differing opinions when it comes to treating relatives [13]. A systematic review by Scarff and Lippmann (2012) indicates that physicians are aware of the complex ethical issues associated with treating relatives. Factors influencing their decision are the involvement of other colleagues, guidelines or legal regulations, and the relative’s clinical situation. The latter is determined mainly by the severity of the disease, the urgency of treatment, the availability of other physicians, and whether the condition falls within the physician’s specialisation [14]. Similar to this, the results of a qualitative study among 449 GPs in the Netherlands described the process of deciding for or against treating a family member: The GP first got a general overview before identifying what disease was involved and in what kind of setting the treatment should be provided. Other common considerations were: The risk of disrupting the actual doctor-patient relationship, the level of confidence in one’s own medical knowledge/skills, an assessment of the consequences of possible treatment errors, the importance of work-life balance, and the impact on the relationship with the relative [15]. Focusing not only on factors influencing the decision whether to treat family members, but also on the consequences associated with it, Reagan et al. (1994) showed that treatment of family members might be burdensome for GPs. According to their cross-sectional study among more than 2000 ambulatory care physicians from Oregon, physicians were concerned that they may not have obtained a full medical history or performed physical examinations in full, and were unsure as to whether they adequately assessed their relatives’ complaints [16].

Whereas treatment of family members is controversially discussed in other countries, in Germany there has hardly been any discussion of aspects that need to be considered when treating relatives [17]. As a consequence, little is known about GPs’ treatment of family members in Germany. Against this backdrop, our study aimed to generate empirical data for Germany and thereby (1) describe GPs’ treatment of relatives, with a comparison of those registered and unregistered as patients in the own practice (2) identify GP characteristics influencing the frequency of treating relatives, and (3) assess the most common reasons for and against treating relatives.

## Methods

This paper is reported in accordance with the STROBE statement [18].

### Study design

The study was designed as a cross-sectional survey.

### Setting and participants

The survey was conducted by the Institute of General Practice, Heinrich-Heine-University Düsseldorf, Germany, and addressed GPs located in the North Rhine region in Germany. Based on the publicly accessible register of the Association of Statutory Health Insurance Physicians North Rhine, all physicians listed as GPs were eligible for participation. This included also some paediatric specialists. Physicians working in the inpatient health sector or in specialised outpatient facilities and physicians working solely as psychotherapists were excluded. In Germany, the specialist training for a GP lasts 5 years (containing 2 years in general medicine in outpatient primary care and 1 year in internal medicine in acute inpatient care). The treatments conducted by GPs cover all aspects of family medicine except for gynaecology. In urban areas treatment of children is often delegated to paediatrics. In addition, 40 practices which were recruited for pilot testing were excluded from the main study.

### Sampling and recruitment

In June 2018, a random sample of 1000 physicians fulfilling the inclusion criteria was invited to participate. When inviting GPs, a stratified approach regarding sex and practice location was used so that the sample consisted of an equal number of male and female GPs established in practices located in rural and urban regions. Due to this approach the sample invited was representative for the North Rhine region with regard to sex, while GPs practicing in rural areas were overrepresented (only about 40% of the practices are considered rural in Germany [19]). This allowed us to take a closer look on the differences in treating relatives in terms of practicing in rural and urban areas. All GPs received the study documents via post. The postal invitation included written information, the questionnaire and a stamped return envelope. Participants were also given the opportunity to return the completed questionnaires via fax.

### Variables and data sources

The written questionnaire was developed by means of a multilevel approach according to Pentzek et al. (2012) [20] and to recommendations of the Institute for Quality Assurance and Transparency in Health Care (2017) [21]. First, a systematic literature search, focus groups and interviews with GPs were conducted in order to explore relevant topics to be assessed in the survey. These topics were reflected upon and amended with experts before drafting a first questionnaire version. The draft questionnaire was pre-tested cognitively and pilot tested with GPs to further develop and optimise it.

The final 7-page questionnaire consisted of 87 items. Among others, it addressed

A) The frequency, content, place and documentation of treating family members during the last 12 months (stratified for spouse/life partner, children, parents and spouse’s parents);
B) Sociodemographic characteristics of the participants;
C) Reasons for and against treating family members in the past on a 6-point Likert scale ranging from 0 (“never the reason for treatment”) to 5 (“very often the reason for treatment”) or rather 0 (“never the reason against treatment”) to 5 (“very often the reason against treatment”) with one free-text field in each case for adding further reasons for and against treating family members;
D) Free-text field for additional comments.

### Bias

In order to minimise the risk of bias due to study non-response and to generate reliable results, several strategies were applied to achieve a sufficient sample size: Assuming a response rate of 30%, 1000 GPs were invited to achieve a sample size of at least 300. All GPs received the study documents via post in conspicuous colourful envelopes. The study documents contained an invitation letter that personally addressed the GPs and defined a deadline for returning the completed questionnaire. A stamped return envelope was provided for returning the questionnaire to the study centre. In case of a response rate < 20% after 10 days, a written reminder was envisaged, but was not necessary. Non-responder interviews were not conducted.

### Statistical methods

In order to describe GPs’ treatment of family members, reasons for and against it, as well as the well-being associated with it, descriptive statistics were applied for quantitative data. Free-text answers were categorised inductively. Percentages and mean values for all variables are reported for valid cases only. Stratified by type of family member, differences regarding treatment of relatives registered at the GPs practice and those not registered as patient at the GPs practice were determined using Pearson’s chi-squared test (Fisher’s exact test if cells were < 5). The nominal significance level for these bivariate analyses was defined as *p* < 0.05. The valid cases and response rate differs among single variables as only GPs having a certain type of relative answered the related questions.

Beside these descriptive analyses, linear regressions were performed to identify what GP characteristics influence the frequency of treating the spouse/life partner, own children, parents and the spouse’s parents. The dependent variable was a sum score ranging from 0 to 20, which was calculated on the basis of the frequency of ten different treatments (for list of the 10 treatments see Tables 2, 3, 4 and 5) during the last 12 months per family member (0 = treatment was not provided; 1 = treatment was provided once or twice, 2 = treatment was provided more than twice). Sex (male/female), household (together with the family member/separated from the family member), practice location (rural/urban), age (years) and practical experience (years) were considered as independent variables. Additionally, a multivariable linear regression was performed with the average sum score for all four groups of family members as a dependent variable. The results of all regression analyses are reported as regression coefficient (Beta) with a 95% confidence interval (CI).

All statistical analyses were performed using IBM SPSS Statistics for Windows, Version 25 (Armonk, New York: IBM Corp.).

## Results

### Participants

In total 1000 questionnaires were sent out, of which 13 could not be delivered. Of the 987 questionnaires delivered, 393 evaluable questionnaires were returned (response rate: 39.8%). The mean age of the GPs was 54.68 years (standard deviation: 8.82), 51.4% (*n* = 202) were female, resulting in a sample which is representative for the North Rhine region in terms of age and sex [22]. The average professional experience as a GP amounted to about 19 years. About half of the GPs surveyed practiced in urban settings (*n* = 186, 47.3%), which is less than in North Rhine in general (about 60%) [19]. The other half practiced in rural areas. More details are provided in Table 1.

**Table 1:** Demographics of survey respondents (*N* = 393)

Characteristic	N		
**Age**, mean ± SD (min-max)	387	Years	54.68 ± 8.82 (32–79)
**Sex**, n (%)	393	Female	202 (51.4)
**Practice experience**, mean ± SD (min-max)	377	Years	18.62 ± 10.35 (1–51)
**Practice location**, n (%)	393	Urban	186 (47.3)
		Rural/Suburban	207 (52.7)
**Medical specialization**, n (%)	386	General Practice	337 (87.3)
		Pediatrician	47 (12.2)
		Other	26 (0.7)
**Spouse/life partner**, n (%)	383	Yes	338 (88.2)
**Children**, n (%)	358	No children	50 (14.0)
		1 Child	59 (16.5)
		2 Children	157 (43.9)
		3 children	71 (18.1)
		≥4 children	21 (5.9)
**Parents**, n (%)	375	No parent alive	110 (29.3)
		1 parent alive	151 (40.3)
		2 parents alive	114 (30.4)
**Spouse’s/life partner’s parents**, n (%)	346	No parent alive	129 (37.2)
		1 parent alive	95 (27.4)
		2 parents alive	122 (35.2)

### Description of GPs’ treatment of family members

In total, 380 of the participants (96.7%) had treated a family member in the last 12 months. 83.7% (*n* = 329) even reported being the primary GP of at least one of their relatives. Of the 335 GPs who reported having a spouse, 75.3% stated that the spouse was registered as a patient in their practice (Table 2). 77.8% of the 302 children recorded were registered as patients in the respondent GP’s own practice (Table 3). Lower rates of being an official GP for their own relatives were found for parents (43.1%) and parents-in-law (28.0%) (Table 4 and 5).

#### Contents of care

Most treatments during the last 12 months were provided significantly more often when the GP was also the primary GP of the relatives (Tables 2, 3, 4 and 5). Only wound care, vaccinations and other injections were provided to a similar extent regardless of whether the relative was officially registered in the practice as a patient. Palliative care of relatives was rarely reported in general.

**Table 2:** Frequency, place and documentation of treatment of spouse/life partner – stratified for unregistered and registered at the GP’s practice

**How often did you provide the following types of treatment to your spouse/life partner in the past 12 months?** (*n* = 335)							
	**A) Spouse/life partner not registered at the GP’s practice (*n*** **= 83), n (%)**			**B) Spouse/life partner registered at the GP’s practice (*n*** **= 252), n (%)**			***p*-value** **A vs. B**
	***never***	***1–2 times***	***≥3 times***	***never***	***1–2 times***	***≥3 times***	
Physical examination (*n* = 328)	40 (51.3)	30 (38.5)	8 (10.3)	59 (23.6)	141 (56.4)	50 (20)	**< 0.001**
Technical diagnostics (e.g., ECG, ultrasound, spirometry) (*n* = 330)	68 (86.1)	9 (11.4)	2 (2.5)	137 (54.6)	102 (40.6)	12 (4.8)	**< 0.001**
Arranging or providing laboratory tests (*n* = 327)	59 (77.6)	13 (17.1)	4 (5.3)	46 (18.3)	165 (65.7)	40 (15.9)	**< 0.001**
Prescribing, recommending or dispensing medication (*n* = 330)	31 (39.7)	30 (38.5)	17 (21.8)	13 (5.2)	107 (42.5)	132 (52.4)	**< 0.001**
Wound care (e.g., minor surgical interventions, removing sutures) (*n* = 329)	52 (66.7)	54 (30.8)	2 (2.6)	173 (68.9)	63 (25.1)	15 (6.0)	0.348
Vaccinations (*n* = 328)	44 (57.1)	32 (41.6)	1 (1.3)	109 (43.4)	136 (54.2)	6 (2.4)	0.103
Other injections or intravenous applications (*n* = 328)	74 (96.1)	2 (2.6)	1 (1.3)	195 (77.7)	37 (14.7)	19 (7.6)	**0.001**
Referral to a specialist or admission to hospital (*n* = 328)	69 (89.6)	7 (9.1)	1 (1.3)	87 (34.7)	127 (50.6)	37 (14.7)	**< 0.001**
Issuing medical certificates, notification of illness or reports (*n* = 325)	69 (90.8)	6 (7.9)	1 (1.3)	170 (68.3)	64 (25.7)	15 (6)	**< 0.001**
Palliative care (*n* = 327)	77 (100)	0 (0.0)	0 (0.0)	246 (98.4)	3 (1.2)	1 (0.4)	0.536
**Did you provide the treatments of your spouse/life partner in your doctor’s office?**^a^ (*n* = 313)							
	**A) Spouse/life partner not registered at the GP’s practice**			**B) Spouse/life partner registered at the GP’s practice**			***p*-value** **A vs. B**
	**Yes**	**Partially**	**No**	**Yes**	**Partially**	**No**	***p***
	6 (9.2)	7 (10.8)	52 (80.0)	80 (32.3)	110 (44.4)	58 (23.4)	**< 0.001**
**Did you document the treatments of your spouse/life partner?**^b^ (*n* = 313)							
	**A) Spouse/life partner not registered at the GP’s practice**			**B) Spouse/life partner registered at the GP’s practice**			***p*-value** **A vs. B**
	**Yes**	**Partially**	**No**	**Yes**	**Partially**	**No**	***p***
	14 (21.5)	9 (13.8)	42 (64.6)	159 (64.1)	58 (23.4)	31 (12.5)	**< 0.001**

^a^Analysis considered only participants who reported having a spouse/life partner

^b^Analysis considered only participants who reported having provided at least one treatment

**Table 3:** Frequency, place and documentation of treatment of children stratified for unregistered and registered at the GP’s practice

**How often did you provide the following types of treatment to your child/children in the past 12 months?** (*n* = 302)							
	**A) Child/children not registered at the GP’s practice (*n*** **= 67), n (%)**			**B) Child/children registered at the GP’s practice (*n*** **= 235), n (%)**			***p*-value** **A vs. B**
	***never***	***1–2 times***	**≥*3 times***	***never***	***1–2 times***	**≥*3 times***	
Physical examination (*n* = 295)	22 (36.1)	23 (37.7)	16 (26.2)	45 (19.2)	135 (57.7)	54 (23.1)	**0.007**
Technical diagnostics (e.g., ECG, ultrasound, spirometry) (*n* = 292)	54 (88.5)	7 (11.5)	0 (0.0)	67.5 (156)	66 (28.6)	9 (3.9)	**0.004**
Arranging or providing laboratory tests (*n* = 296)	47 (75.8)	15 (24.2)	0 (0.0)	77 (32.9)	135 (57.7)	22 (9.4)	**< 0.001**
Prescribing, recommending or dispensing medication (*n* = 297)	18 (29.0)	27 (43.5)	17 (27.4)	21 (8.9)	120 (51.1)	94 (40.0)	**< 0.001**
Wound care (e.g., minor surgical interventions, removing sutures) (*n* = 295)	35 (56.5)	23 (37.1)	4 (6.5)	130 (55.8)	86 (36.9)	17 (7.3)	0.974
Vaccinations (*n* = 295)	46 (74.2)	16 (25.8)	0 (0.0)	65 (27.9)	137 (58.8)	31 (13.3)	**< 0.001**
Other injections or intravenous applications (*n* = 294)	60 (96.8)	2 (3.2)	0 (0.0)	220 (94.8)	9 (3.9)	3 (1.3)	0.645
Referral to a specialist or admission to hospital (*n* = 294)	48 (78.7)	12 (19.7)	1 (1.6)	109 (46.8)	102 (44.8)	22 (9.4)	**< 0.001**
Issuing medical certificates, notification of illness or reports (*n* = 294)	49 (79.0)	13 (21.0)	0 (0.0)	97 (41.8)	104 (44.8)	31 (13.4)	**< 0.001**
Palliative care (*n* = 293)	60 (100.0)	0 (0.0)	0 (0.0)	229 (100.0)	0 (0.0)	0 (0.0)	–
**Did you provide the treatments of your child/children in your doctor’s office?**^a^ (*n* = 282)							
	**A) Child/children not registered at the GP’s practice**			**B) Child/children registered at the GP’s practice**			***p*-value** **A vs. B**
	**Yes**	**Partially**	**No**	**Yes**	**Partially**	**No**	***p***
	2 (4.1)	10 (20.4)	37 (75.5)	58 (24.9)	92 (39.5)	83 (35.6)	**< 0.001**
**Did you document the treatments of your child/children?**^b^ (*n* = 281)							
	**A) Child/children not registered at the GP’s practice**			**B) Child/children registered at the GP’s practice**			***p*-value** **A vs. B**
	**Yes**	**Partially**	**No**	**Yes**	**Partially**	**No**	***p***
	14 (28.6)	9 (18.4)	26 (53.1)	135 (58.2)	58 (25.0)	39 (16.8)	**< 0.001**

^a^Analysis considered only participants who reporting having at least one child

^b^Analysis considered only participants who reporting having provided at least one treatment

**Table 4:** Frequency, place and documentation of treatment of parents – stratified for unregistered and registered at the GP’s practice

**How often did you provide the following types of treatment to your parents in the past 12 months?** (*n* = 262)							
	**A) Parent/parents not registered at the GP’s practice (*n*** **= 149), n (%)**			**B) Parent/parents registered at the GP’s practice (*n*** **= 113), n (%)**			***p*-value** **A vs. B**
	***never***	***1–2 times***	**≥*3 times***	***never***	***1–2 times***	**≥*3 times***	
Physical examination (*n* = 251)	80 (57.6)	44 (31.7)	15 (10.8)	10 (8.9)	52 (46.4)	50 (44.6)	**< 0.001**
Technical diagnostics (e.g., ECG, ultrasound, spirometry) (*n* = 251)	128 (92.1)	11 (7.9)	0 (0.0)	35 (31.3)	57 (50.9)	20 (17.9)	**< 0.001**
Arranging or providing laboratory tests (*n* = 252)	122 (87.8)	13 (9.4)	4 (2.9)	11 (9.7)	57 (50.9)	20 (17.9)	**< 0.001**
Prescribing, recommending or dispensing medication (*n* = 252)	59 (42.4)	53 (38.1)	27 (19.4)	2 (1.8)	26 (23.0)	85 (75.2)	**< 0.001**
Wound care (e.g., minor surgical interventions, removing sutures) (*n* = 252)	112 (80.6)	21 (15.1)	6 (4.3)	59 (52.2)	35 (31.0)	19 (16.8)	**< 0.001**
Vaccinations (*n* = 252)	120 (86.3)	18 (12.9)	1 (0.7)	46 (40.7)	65 (57.5)	2 (1.8)	**< 0.001**
Other injections or intravenous applications (*n* = 249)	132 (95.7)	4 (2.9)	2 (1.4)	77 (69.4)	22 (19.8)	12 (10.8)	**< 0.001**
Referral to a specialist or admission to hospital (n = 252)	111 (79.9)	21 (15.1)	7 (5.0)	23 (20.4)	45 (39.8)	45 (39.8)	**< 0.001**
Issuing medical certificates, notification of illness or reports (*n* = 252)	133 (95.7)	5 (3.6)	1 (0.7)	80 (70.8)	27 (23.9)	6 (5.3)	**< 0.001**
Palliative care (*n* = 251)	138 (99.3)	0 (0.0)	1 (0.7)	107 (95.5)	1 (0.9)	4 (3.6)	0.145
**Did you provide the treatments of your parent/parents in your doctor’s office?**^a^ (*n* = 205)							
	**A) Parent/parents not registered at the GP’s practice**			**B) Parent/parents registered at the GP’s practice**			***p*-value** **A vs. B**
	**Yes**	**Partially**	**No**	**Yes**	**Partially**	**No**	**p**
	6 (6.5)	9 (9.8)	77 (83.7)	44 (38.9)	42 (37.2)	27 (23.9)	**< 0.001**
**Did you document the treatments of your parent/parents?**^b^ (*n* = 203)							
	**A) Parent/parents not registered at the GP’s practice**			**B) Parent/parents registered at the GP’s practice**			***p*-value** **A vs. B**
	**Yes**	**Partially**	**No**	**Yes**	**Partially**	**No**	***p***
	23 (25.3)	8 (8.8)	60 (65.9)	84 (75.0)	24 (21.4)	4 (3.6)	**< 0.001**

^a^Analysis considered only participants who reporting having at least one parent

^b^Analysis considered only participants who reported having provided at least one treatment

**Table 5:** Frequency, place and documentation of treatment of spouse’s parents – stratified for unregistered and registered at the GP’s practice

**How often did you provide the following types of treatment to your spouse’s/life partner’s parents in the past 12 months?** (*n* = 214)							
	**A) Spouse’s/life partner’s parents not registered at the GP’s practice (*n*** **= 154), n (%)**			**B) Spouse’s/life partner’s parents registered at the GP’s practice (*n*** **= 60), n (%)**			***p*-value** **A vs. B**
	***never***	***1–2 times***	**≥*3 times***	***never***	***1–2 times***	**≥*3 times***	
Physical examination (*n* = 201)	107 (75.9)	29 (20.6)	5 (3.5)	4 (6.7)	30 (50.0)	26 (43.3)	**< 0.001**
Technical diagnostics (e.g., ECG, ultrasound, spirometry) (*n* = 201)	132 (93.6)	9 (6.4)	0 (0.0)	12 (20.0)	37 (61.7)	11 (18.3)	**< 0.001**
Arranging or providing laboratory tests (*n* = 201)	128 (90.8)	11 (7.8)	2 (1.4)	6 (10.0)	61.7 (37)	11 (18.3)	**< 0.001**
Prescribing, recommending or dispensing medication (*n* = 201)	96 (68.1)	25 (24.8)	10 (7.1)	0 (0.0)	15 (25.0)	45 (75.0)	**< 0.001**
Wound care (e.g., minor surgical interventions, removing sutures) (*n* = 201)	124 (87.9)	15 (10.6)	2 (1.4)	41 (68.3)	15 (25.0)	4 (6.7)	**0.003**
Vaccinations (*n* = 201)	125 (88.7)	16 (11.3)	0 (0.0)	23 (38.3)	35 (58.3)	2 (3.3)	**< 0.001**
Other injections or intravenous applications (*n* = 201)	138 (97.9)	3 (2.1)	0 (0.0)	46 (76.7)	11 (18.3)	3 (5.0)	**< 0.001**
Referral to a specialist or admission to hospital (*n* = 201)	124 (87.9)	17 (12.1)	0 (0.0)	15 (25.0)	21 (35.0)	24 (40.0)	**< 0.001**
Issuing medical certificates, notification of illness or reports (*n* = 200)	136 (96.5)	5 (3.5)	0 (0.0)	37 (62.7)	19 (32.2)	3 (5.1)	**< 0.001**
Palliative care (*n* = 201)	141 (100)	0 (0.0)	0 (0.0)	58 (96.7)	2 (3.3)	0 (0.0)	**0.029**
**Did you provide the treatments of your spouse’s/life partner’s parent/parents in your doctor’s office?**^a^ (*n* = 121)							
	**A) Parent/parents not registered at the GP’s practice**			**B) Parent/parents registered at the GP’s practice**			***p*-value** **A vs. B**
	**Yes**	**Partially**	**No**	**Yes**	**Partially**	**No**	***p***
	8 (13.1)	7 (11.5)	46 (75.5)	29 (48.3)	19 (31.7)	12 (20.0)	**< 0.001**
**Did you document the treatments of your spouse’s/life partner’s parent/parents?**^b^ (*n* = 120)							
	**A) Parent/parents not registered at the GP’s practice**			**B) Parent/parents registered at the GP’s practice**			***p*-value** **A vs. B**
	**Yes**	**Partially**	**No**	**Yes**	**Partially**	**No**	***p***
	20 (32.8)	7 (11.5)	34 (55.7)	53 (89.8)	3 (5.1)	3 (5.1)	**< 0.001**

^a^Analysis considered only participants who reported having at least one spouse’s/life partner’s parent

^b^Analysis considered only participants who reported having provided at least one treatment.

An analysis of the treatment of family members not officially registered as patients in the GPs’ practices revealed that prescribing, recommending or dispensing pharmacotherapy were among the most common care services provided (Tables 2, 3, 4 and 5). In addition, treatment of unregistered spouses/life partners most frequently involved physical examinations (48.8%) and vaccinations (42.9%), whereas referral to a specialist or admission to hospital (10.4%), issuing a medical certificate (9.2%) and administration of other injections or intravenous treatments (8.9%) were seldom reported (Table 2). Treatment of children (Table 3), parents (Table 4) and spouse’s/life partner’s parents (Table 5) not officially registered as patients was less common overall than treatment of spouses/life partners. For unregistered children and parents, the most frequent content of care after pharmacotherapy were physical examinations (children: 63.9%, parents: 42.5%). Additionally, wound care was frequently reported for children (43.6%), while the only frequently provided care contents for the parents of the spouse/life partner was pharmacotherapy.

#### Treatment place

Relatives who were registered patients were treated more often in the doctor’s office than those who were not officially registered. Non-registered relatives were predominantly treated outside the practice premises (75.5 to 80%). However, as many as 20 to 35.6% of the treatments of registered relatives were not provided in the practice (Tables 2, 3, 4 and 5).

#### Treatment documentation

Only one half of treatments provided to unregistered family members was documented by the GPs (53.1 to 65.9%). For children and spouses/life partners registered in the practice, treatments remained undocumented in 12.5% (spouses/life partners) and 16.8% (children) of all cases, whereas treatment of registered parents (3.6%) and parents of spouses/life partners (5.1%) remained undocumented less frequently. The data revealed that there is a particular lack of treatment documentation for relatives living in the same household. More details are provided in Tables 2, 3, 4 and 5.

### Factors influencing the treatment of family members

The linear regression analyses revealed that the sum score representing the treatment frequency of different family members was influenced by various factors. We checked for the influencing factors sex, joint household, practice experience, age and practice location (rural/urban). More details are provided in Table 6.

**Table 6:** Factors influencing the frequency of treating family members

a) Spouse/life partner (*n* = 306)			
Independent variable	**Beta**	**95% CI**	***p*-value**
Male gender	1.12	0.34–1.90	0.005*
Joint Household	1.52	0.21–2.83	0.023*
Practice experience	0.04	−0.03 – 0.12	0.231
Age	0.03	−0.05 – 0.12	0.468
Urban practice location	−0.21	− 0.93 – 0.51	0.566
b) Own children (*n* = 280)			
Independent variable	**Beta**	**95% CI**	***p*-value**
Joint household with ≥1 child	1.09	0.18–2.00	0.019*
Urban practice location	−0.57	−1.29 – 0.16	0.125
Practice experience	0.06	−0.01 – 0.13	0.089
Male gender	−0.32	− 1.10 – 0.45	0.421
Age	−0.03	−0.12 – 0.06	0.484
c) Own parents (*n* = 235)			
Independent variable	**Beta**	**95% CI**	***p*-value**
Practice experience	0.12	0.01–0.23	0.028*
Male gender	0.99	−0.17 – 2.15	0.094
Urban practice location	−0.84	−1.95 – 0.26	0.132
Age	−0.04	−0.17 – 0.08	0.506
Joint household with ≥1 parent	0.33	−1.34 – 2.00	0.698
d) Spouse’s parents (*n* = 190)			
Independent variable	**Beta**	**95% CI**	***p*-value**
Male gender	2.36	1.20–3.53	< 0.001*
Age	0.09	−0.04 – 0.22	0.154
Practice experience	−0.06	−0.18 – 0.05	0.271
Urban practice location	−0.50	−1.59 – 0.60	0.371
Joint household with ≥1 spouse parent	1.50	−2.28 – 5.28	0.435

* significant considering a 95% confidence interval

#### Spouse/life partner

An analysis of the data of 306 GPs showed that the frequency of treating the spouse/life partner was higher when the GP was male (Beta: 1.12 [95% CI: 0.34–1.90], *p* = 0.005) and lived with his/her spouse/partner (Beta: 1.52 [95% CI: 0.21–2.83], *p* = 0.023).

#### Own children

With regard to the treatment of own children, an analysis based on the data of 280 GPs identified a conjoint household as a single factor significantly influencing the treatment frequency (Beta: 1.09 [95% CI: 0.17–2.00], *p* = 0.019).

#### Own parents

Analyses based on the data of 235 GPs revealed that the frequency of treating the own parents was slightly influenced by the GPs’ practical experience (Beta: 0.12 [95% CI: 0.01–0.23], *p* = 0.028).

#### Spouse’s parents

An analysis of the frequency of treating the spouse’s parents (*n* = 190) demonstrated that being male was a highly significant influencing factor (Beta: 2.36 [95% CI: 1.20–3.53], *p* < 0.001).

#### Any family member

An additional multivariable regression based on the data of 372 GPs revealed three independent predictors that significantly increased or decreased the frequency of treating any family member: male gender (Beta: 0.82 [95% CI: 0.23–1.41], *p* = 0.006), practice located in urban region (Beta: − 0-64 [95% CI: − 1.19- -0.09], *p* = 0.022) and higher age (Beta: 0.05 [95% CI: 0.02–0.08], *p* = 0.004).

### Reasons for treating family members

Of the reasons for treating family members assessed in the questionnaire, the following three achieved the highest mean values on the scale from 0 (“never the reason”) to 5 (“very often the reason”): Practical advantages for relatives (4.32), great confidence of relatives in the GP and his/her medical skills (4.18), and relatives being only mildly ill as well as the necessary treatment being manageable (3.93).

The most important reason for deciding against treatment was that the treatment needed was outside the GPs’ area of expertise/routine (3.33). For all reasons addressed in the questionnaire, see Table 7.

**Table 7:** Reasons for and against treating family members

Reasons for treatment	n	Mean ± SD	Reasons against treatment	n	Mean ± SD
Practical advantages for your relatives (e.g., waiting time reduction, logistics)	383	4.32 ± 1.34	The treatment was outside your specialisation/routine	374	3.33 ± 1.63
Great confidence of your relatives in you and in your medical skills	381	4.18 ± 1.14	Difficulty in remaining objective during treatment	375	1.98 ± 1.79
Your relatives were only slightly ill and the necessary treatment was manageable	383	3.93 ± 1.36	The relatives were very seriously ill	368	1.55 ± 1.83
Your relatives expect you to be available for medical treatment	385	3.71 ± 1.59	The relatives should have looked for or consulted their own GP	367	1.39 ± 1.81
You know your relatives and can deal with individual cases of illness better than other doctors.	382	3.55 ± 1.55	The relatives would not have listened to medical advice	369	0.94 ± 1.37
Emergency	379	2.77 ± 1.93	Family conflicts	374	0.48 ± 1.11
Your relatives asked you for a second opinion	385	2.38 ± 2.01			
You were travelling with your relatives	381	2.07 ± 1.91			
Your relatives only wanted to be treated by you and would have been harmed otherwise	379	1.57 ± 1.87			
No other GP was available (e.g., in a rural region)	380	0.54 ± 1.26			

Inductive categorisation of the free-text answers showed that the most important additionally mentioned reasons for and against treatments were also of a practical nature, i.e., associated with less organisational effort (for), but also less objective decisions (against). The factors trust, financial aspects and dissatisfaction with the treatment of another GP were also mentioned as reasons for treating relatives.

## Discussion

Nearly all GPs participating in the survey had treated a family member in the last 12 months, most commonly by prescribing or recommending medications and providing physical examinations. About 84% of the GPs had treated at least one registered relative. All types of treatment were performed more frequently for those relatives who were registered as patients in the GPs’ practice. It is to be expected that relatives will be treated more frequently in the practice rooms if they are registered as patients. However, even in this group, treatments outside the practice rooms occur in relevant numbers. The same applies to documentation, which is very rarely carried out when treating unregistered relatives and is by no means consistently carried out when treating registered relatives.

The medical treatment of one’s own family members, regardless of their registration status, can lead to role conflicts when the GP has to make decisions as a doctor on the one hand and as a possibly concerned relative such as a father/mother, son/daughter or husband/wife on the other. The main reasons for treatment of own relatives were practical advantages for the relatives like decreasing the waiting time. In general, such individual, practical reasons for treatment appear to overweigh possible concerns of GPs about appropriateness of treating own relatives. Especially when family members were not officially registered as patients or lived in the same household, treatment was seldom provided in the practice and documented.

Overall, as a GP being male and of higher age was associated with more frequent treatment of relatives. The results from this survey among German GPs are comparable to those from previous international studies among GPs and other professions. Several studies revealed that informal treatment of own relatives is common practice [23, 24], especially among GPs and paediatricians [16, 25, 26]. Treatment of the partner and own children is most common [27, 28], which might be explained by a study that revealed that treating persons living in the same household feels natural and is uncomplicated for physicians [29]. In addition, GPs in different studies reported feeling more comfortable with treating their children compared to treating other relatives [16, 30]. Irrespective of the relationship to the family member and the living situation, treatment of relatives was found to be more common among male physicians [16], which is consistent with the results of the present study.

Regarding the content of care, several studies underline that treatment of own relatives includes, in particular, recommending and prescribing medications as well as performing physical examinations [16, 24, 26, 31]. As outlined by the participants in the present study, these treatments are provided mainly for practical reasons [14, 16, 26] and because the family members might have more confidence in a physician they know personally [14]. Like in our study, treatment demands outside the GPs’ routine and difficulties in remaining neutral are key reasons for rejecting the treatment of family members [14, 31, 32]. Interestingly, even though some authors raise ethical concerns about insufficient treatment documentation [13], documentation seems to be neglected when treating relatives [27].

### Limitations

Overall, nearly 40% of all physicians who were invited to participate completed the questionnaire. Compared to other studies in the primary care setting, this response rate is quite high and thus a key strength of the study.

However, there are some potential limitations: As no incentives were provided, GPs who were more motivated or who have particular reservations about treating family members might be overrepresented in the sample. Ethnic origin may also have an influence on treatment of relatives; this variable was not surveyed. In addition, GPs who do not have relatives or who live far away from their family might not have felt addressed by the survey and might be underrepresented. Therefore, selection bias cannot be excluded. Moreover, the study addressed GPs located only in the North Rhine region and used a stratified approach regarding sex and practice location when inviting GPs. While gender proportions in our sample are representative of the North Rhine region, as a result of stratification more GPs from rural areas (52.7%) participated in the survey than from urban areas, even though only about 40% of practices are located in rural areas. However, no influence of practicing in a rural area on treating relatives was found. Furthermore this study followed an exploratory approach in order to get insights into GPs treatment of relatives in Germany, multiple testing was present during analyses and some significant findings might be due to chance.

## Conclusions

Based on the results of this study we conclude that treatment of physician’s relatives represents a subject of high everyday relevance in Germany, since nearly all GPs in primary care are involved in the treatment of family members. This includes informal medical advice and treatment of relatives’ at home but also regular medical care of family members who are registered in the GPs’ practices. We found that male and experienced GPs are more involved in the treatment of relatives than their female and less experienced colleagues.

Frequent at-home treatments and low documentation rates of treatment of relatives could be signs of deviations from the professional routine. Further research on the quality of care in families of physicians is necessary to assess potential risks due to deviations from the routine. Some of the main reasons for treating own relatives are practical considerations (e.g., time savings) and explicit or implicit expectations of family members which may lead to role conflicts for the physicians. To better understand this issue, further research on the views and beliefs of physicians and family members is required. Furthermore, we conclude that the issue of treating one’s relatives should be addressed in undergraduates’ medical training, vocational training and continuing medical education. Moreover, it should also be included in the Medical and Ethical Guidelines for Physicians.

## Data Availability

The datasets analysed within the scope of this study are available from the corresponding author on reasonable request.
